# Tuberous sclerosis with visceral leishmaniasis: a case report

**DOI:** 10.4076/1752-1947-3-9027

**Published:** 2009-09-08

**Authors:** Krishna Pandey, Prabhat K Sinha, Vidyanand R Das, Nawin Kumar, Sanjiva Bimal, Rakesh B Verma, Neena Verma, Chandra S Lal, Roshan K Topno, NA Siddiqui, Dharmendra Singh, Pradeep Das

**Affiliations:** 1 Department of Clinical Medicine, Rajendra Memorial Research Institute of Medical Sciences (Indian Council of Medical Research), Agamkuan, Patna - 800 007, Bihar, India; 2 Department of Immunology, Rajendra Memorial Research Institute of Medical Sciences (Indian Council of Medical Research), Agamkuan, Patna - 800 007, Bihar, India; 3 Department of Pathology, Rajendra Memorial Research Institute of Medical Sciences (Indian Council of Medical Research), Agamkuan, Patna - 800 007, Bihar, India; 4 Department of Biochemistry, Rajendra Memorial Research Institute of Medical Sciences (Indian Council of Medical Research), Agamkuan, Patna - 800 007, Bihar, India; 5 Department of Epidemiology, Rajendra Memorial Research Institute of Medical Sciences (Indian Council of Medical Research), Agamkuan, Patna - 800 007, Bihar, India; 6 Department of Molecular Biology, Rajendra Memorial Research Institute of Medical Sciences (Indian Council of Medical Research), Agamkuan, Patna - 800 007, Bihar, India

## Abstract

**Introduction:**

Visceral leishmaniasis, a tropical infectious disease, is a major public health problem in India. Tuberous sclerosis, a congenital neuro-ectodermosis, is an uncommon disease which requires life long treatment.

**Case presentation:**

A 15-year-old Indian patient, presented to the outpatient department of our institute with a high-grade fever for two months, splenomegaly and a history of generalized tonic-clonic convulsions since childhood. The clinical and laboratory findings suggested visceral leishmaniasis with tuberous sclerosis. The patient was treated with miltefosine and antiepileptics.

**Conclusion:**

The patient responded well and in a follow up six months after presentation, she was found free of visceral leishmaniasis and seizures. Diagnosis and treatment of this rare combination of diseases is difficult.

## Introduction

*Leishmania donovani*, is a protozoan parasite that causes visceral leishmaniasis (VL) in India. The disease is transmitted by the bite of infected female sand flies (*phlebotomus argentipes*). About 350 million people are at risk worldwide with an estimated prevalence of 2.5 million and incidence of 0.5 million new cases every year. India, Nepal, Bangladesh, Sudan and Brazil account for 90% of the cases worldwide. Bihar accounts for about 80% of the total Indian cases. The disease affects those from a lower socio-economic background and it is estimated that about 90% of people with VL earn less than $2 a day [1].

Tuberous sclerosis is an example of a phakomatosis as are neurofibromatosis, Sturge-Weber syndrome and Von Hippel-Lindau disease, among others. These diseases are known to have autosomal dominant hereditary transmission as well as involvement of organs of ectodermal origin, such as the nervous system, the eyes and the skin. Slow evolution of lesions in childhood and adolescence, tendency to form hamartomas and a pre-disposition to malignant transformation are also characteristic. The disease is transmitted as an autosomal dominant trait and has a prevalence of 1 in 20,000 to 1 in 30,000 [2].

## Case presentation

A 15-year-old girl of Indian origin born to non-consanguineous parents presented to our outpatient department in Feb. 2007. She had two younger brothers who were asymptomatic. She presented with fever of 39°C along with chills of two months duration with weakness and malaise. The fever had not responded to antimalarials or antibiotics. The patient also complained of repeated episodes of generalized tonic-clonic convulsions since childhood. She had multiple acne-like spots on the cheeks. The patient's mother complained that the child was very weak in studies and had failed to pass class eight at school three times. On clinical examination, her weight was 45 kg, pulse rate 120/min, respiratory rate 22/min and blood pressure 110/70 mmHg in the left arm in the supine position. She appeared pale but had no jaundice, cyanosis, clubbing or lymphadenopathy. Chest examination and cardiovascular system examination were normal. An abdominal examination revealed the liver to be about 2 cm and the spleen about 4 cm below the respective costal margin in the mid-axillary line.

The patient's face was covered with acne like patches (adenoma sebaceum) which had persisted for the last 12 years. The patient's mother apparently thought that this might have been due to an allergic drug reaction. Her back was examined for a shagreen patch and freckles were present in the lumbosacral region. The neurological examination was otherwise normal.

## Laboratory examination

The total count was 4200/mm^3^ with hemoglobin of 7.5 gm/dl. The platelet count was 100,000/mm^3^. The renal and liver function tests were within normal limits. The prothrombin time was 3 seconds above the normal limit of control of 12 seconds. Splenic aspiration was performed and amastigotes of *Leishmania donovani* (LD, 2+ according to WHO criteria) were found. A chest X-ray of the posteroanterior view, an electrocardiogram and echocardiography were normal. Ultra sonography of the abdomen showed hepatosplenomegaly. Electroencephalogram showed irregular dysrhythmic bursts of high voltage spikes and slow waves bilaterally. Computed tomography (CT) scan of the brain showed multiple subependymal periventricular calcified lesions. A chromosomal examination was not carried out. The eye examination conducted by an ophthalmologist was normal.

Considering the above facts, and the clinical triad of mental retardation, epilepsy and adenoma sebaceum, a diagnosis of tuberous sclerosis with visceral leishmaniasis was made. Treatment was initiated with miltefosine capsules (50 mg) twice daily after meals for 28 days. She was also started on iron supplements and 300 mg tablets of the anti-epileptic phenytoin sodium at bed time were prescribed. After one month her platelet count was normal. The spleen regressed and no LD bodies were demonstrated in the bone-marrow aspirate. She had no fever and appeared cheerful. The patient was told to continue taking iron and folic acid supplements along with phenytoin sodium. One month after presentation, there was no fever; her spleen and liver were not palpable and she did not have any seizures. She was told to continue taking phenytoin sodium, indefinitely. The patient was free from VL and seizures at six month follow-up.

## Discussion

Our case report describes a rare combination of tuberous sclerosis and visceral leishmaniasis. VL has a high prevalence in India, particularly in eastern states like Bihar, Bengal and Eastern Uttar Pradesh. The diagnosis and management of this disease is difficult. However, newer diagnostic tools like the rK39 strip test (which has a sensitivity and specificity close to 98%) can be applied in field conditions [3]. This, along with splenic and bone marrow aspiration and other sophisticated tests like the direct agglutination test (DAT) and nested polymerase chain reaction (PCR) can be help in making a diagnosis.

Regarding management, sodium antimony gluconate is developing resistance and a response rate of less than 40% has been reported in Bihar [4]. Pentamidine use has been stopped of late particularly because of multiple toxic effects such as diabetes and anaphylactic shock. Amphotericin B can be an effective drug but requires hospital admission and monitoring particularly for hypokalaemia and nephrotoxicity.

Miltefosine can be given orally and has a cure rate of about 95% with limited toxicity - mainly gastrointestinal according to the observations of Phase III and Phase IV trials at our institute and other clinical centres of Bihar [5,6]. Sitamaquine, another oral drug, is being tried but is still at Phase II. Injectable aminoglycosides, namely paromomycin, has completed Phase III trial and its Phase IV clinical trial is in progress. This drug has a cure rate of about 95% [7]. Miltefosine and Amphotericin B appear to be the best available drugs for VL therapy at present. Miltefosine has been recommended for use in the VL elimination program in India at pilot level by the Central Government. However, being a teratogenic drug, its use is limited in pregnant women.

Tuberous sclerosis is an autosomal dominant disorder caused by mutation in either of the two genes TSC1 (located on chromosome 9q34) or TSC2 (located on 16p 13.3). In about 50% of these cases there are grey or yellow plaques (gliomatous tumors) in the retina or optic disc (phakoma), as well as renal hamartomas. Rhabdomyomas are present in the heart in at least 50% of cases. In about 90% of cases, congenital hypomelanotic macules, or "ash leaf" lesions, can occur on the skin which can be identified by Wood's lamp examination. About 90% of these patients present with adenoma sebaceum on the cheek with a shagreen patch on the lumbosacral region.

The diagnosis was based on the above mentioned features, as well as family history although chromosomal analysis was not done. Death often occurs due to conduction defects in the heart, intractable epilepsy and renal failure or malignant transformation. The TSC2 gene has been associated with polycystic kidney disease. Positron emission tomography (PET) scanning with 2-deoxy-2-[F-18]fluoro-D-glucose can assess the full extent of functional brain abnormality in tuberous sclerosis [8]. The use of alpha (C) methyl-L-tryptophan PET has proved to be a useful tool in the identification of epileptogenic tubers and improves the outcome of surgery for epilepsy in tuberous sclerosis [9].

Various drugs have been used to treat epilepsy but the disease is usually resistant. Levetiracetam was found to have good results [10]. Antiepileptic treatment has to be taken indefinitely. A multi-disciplinary treatment approach has to be undertaken for lesions in the other organs like the kidneys, heart and eyes including surgery for cerebral tubers and subependymal nodules. The drug interactions between various antileishmanial drugs, in this case miltefosine, with antiepileptics like phenytoin sodium (potential enzyme inducers) need to be taken into consideration.

## Conclusion

The diagnosis and management cost of this combination of diseases is beyond the means of poorer patients, particularly because treatment has to be given for life, especially in case of intractable epilepsy. This can require costly stereotactic brain surgery.

**Figure 1:**
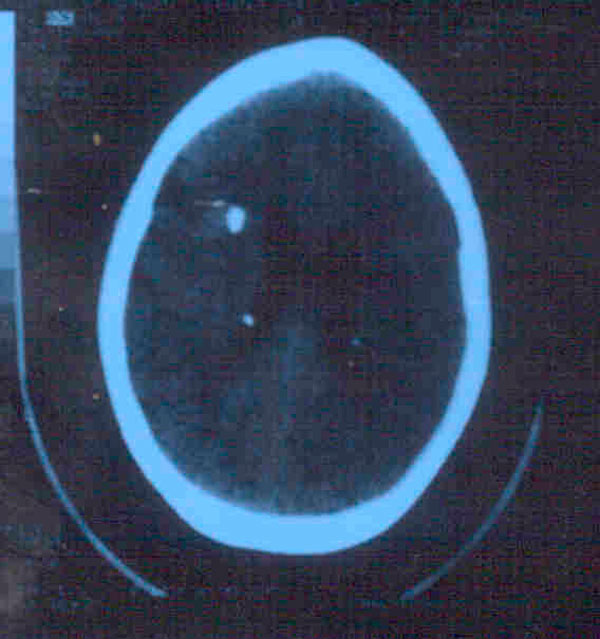
**Computerized tomography (CT) of the brain showing bilateral subependymal periventricular tubers**.

## Abbreviations

CT: computed tomography; DAT: direct agglutination test; LD: *Leishmania donovani*; PCR: polymerase chain reaction; PET: positron emission tomography; VL: visceral leishmanisis.

## Consent

Written informed consent for publication of this case report and accompanying images was obtained from the patient's father, as the patient was a minor. A copy of the written consent is available for review by the Editor-in-Chief of this journal.

## Competing interests

The authors declare that they have no competing interests.

## Authors' contributions

KP recorded the patient data and performed clinical examination and management of the patient. PKS, VNRD, NK and RKT helped in case management. SB, NV, CSL and DS did the immunological, pathological, biochemical and molecular profile of the patient respectively. RBV, NAS and PD were the major contributors in writing the manuscript. All authors read and approved the final manuscript.
